# Oral Health, Nutritional Choices, and Dental Fear and Anxiety

**DOI:** 10.3390/dj5010008

**Published:** 2017-01-21

**Authors:** Jennifer R. Beaudette, Peter C. Fritz, Philip J. Sullivan, Wendy E. Ward

**Affiliations:** 1 Department of Kinesiology, Brock University, St. Catharines, ON, L2S 3A1, Canada; jb10im@brocku.ca (J.R.B.); drpeterfritz@me.com (P.C.F.); psullivan@brocku.ca (P.J.S.); 2 Reconstructive Periodontics and Implant Surgery Clinic, Fonthill, ON L0S 1E5, Canada

**Keywords:** anxiety, fear, nutrition, oral health, periodontal

## Abstract

Oral health is an integral part of overall health. Poor oral health can lead to an increased risk of chronic diseases including diabetes mellitus, cardiovascular disease, and some types of cancer. The etiology of these diseases could be linked to the individual’s inability to eat a healthy diet when their dentition is compromised. While periodontal or implant surgery may be necessary to reconstruct tissue around natural teeth or replace missing teeth, respectively, some individuals avoid such interventions because of their associated fear and anxiety. Thus, while the relationship between poor oral health, compromised nutritional choices and fear and anxiety regarding periodontal procedures is not entirely new, this review provides an up-to-date summary of literature addressing aspects of this complex relationship. This review also identifies potential strategies for clinicians to help their patients overcome their fear and anxiety associated with dental treatment, and allow them to seek the care they need.

## 1. Introduction

This review will identify what is known about the complex relationship among poor oral health, compromised nutritional choices, and fear and anxiety regarding periodontal procedures. Specifically, tooth loss and how it affects food and/or nutrient intake will be discussed as well as how dental fear and anxiety can lead patients to avoid dental treatment, thereby potentially further compromising their oral health through an inability to consume a healthful diet. Some strategies to help patients manage their dental anxiety are briefly discussed. The articles cited in this literature review were found by searching a number of online databases including Medline, Web of Science Complete, and Academic Search Complete. Examples of some of the search terms used in combination are: oral health, nutrition, nutrient intake, dental status, dental fear, anxiety and periodontal. 

Nutrition and oral health are inextricably linked. Poor oral health can affect an individual’s ability to eat certain nutritious foods while poor nutrition can increase an individual’s risk of poor oral health including periodontal disease and tooth loss. Periodontal disease has been linked to diabetes mellitus, cardiovascular disease, and some types of cancer [1,2,3]. Tooth loss, which may or may not be related to periodontal disease, has been associated with an increased risk of a number of chronic diseases, including coronary heart disease and chronic kidney disease [4,5]. It may also be associated with poor nutrient intake. Epidemiological evidence suggests a diet high in fruits, vegetables, and whole grains can decrease the risk of coronary heart disease [6]. Moreover a diet high in vitamin C, which is found in many of the fruits and vegetables that individuals with poor oral health might find difficult to eat, is protective against some types of cancers, including of the mouth [6]. Thus, poor nutrient intake that originates from compromised dental status may result in a higher risk of chronic disease.

**Figure 1:**
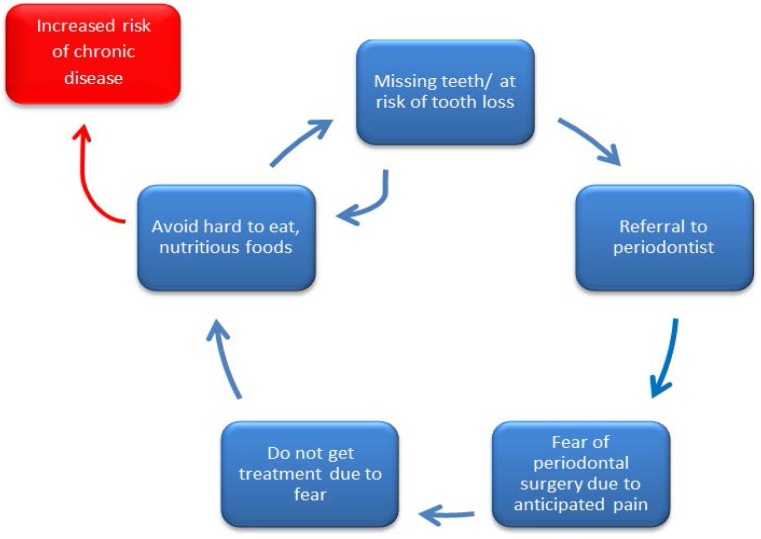
It is hypothesized that individuals can find themselves cycling between poor oral health (missing teeth or at risk of tooth loss) and poor nutrition (avoidance of hard to eat, nutritious foods) because they fear the necessary dental treatment that can break the cycle. Remaining in the cycle between poor oral health and poor nutrition may put an individual at an increased risk of developing chronic disease.

Replacing and maintaining natural teeth improves masticatory ability, allowing individuals to consume a varied and nutritious diet, and is a strategy to end the cycle that can set an individual on a trajectory for chronic disease development (Figure 1). However, while reconstructive periodontal surgery and/or implant placement can avoid limiting food choices due to poor dentition, many people refuse or delay these procedures because of fear and anxiety (Figure 1). Identifying and implementing strategies to reduce fear and anxiety among patients could end the cycle by encouraging more patients to seek necessary treatment; leading to greater oral and overall health. 

## 2. Tooth Loss 

The Oral Health Module of the Canadian Health Measures Survey revealed that 6.4% of Canadian adults (aged 20–79) are edentulous [7]. When broken down by age groups, the highest rate of edentulism was among older adults (aged 60–79) at 21.7% compared to the 40–59 year old age group with a rate of 4.4%. A full complement of teeth is considered to be 28 teeth, although an individual can have up to 32 teeth if there is no tooth loss and all four third molars are present. The average number of teeth that Canadian adults have is 24.53 teeth. Of the 93.6% of dentate Canadian adults, 42.3% have all 28 teeth, 36.7% have between 28 and 21 teeth, and 14.6% have fewer than 21 teeth [7]. A total of 57.7% of Canadian adults are missing one or more teeth and an impaired dentition can lead to negative health outcomes [7]. 

Data from the Global Burden of Disease study showed that in 2010, 3.9 billion people were affected by oral health conditions [8]. Untreated dental caries of permanent teeth were the most prevalent of these conditions, affecting 35% of the global population. Severe periodontitis had a global prevalence of 11% and severe tooth loss (<9 remaining teeth) had a global prevalence of 2% [8]. In the US, the prevalence of periodontitis was assessed by the National Health and Nutrition Examination Survey (NHANES) [9]. Beginning in 2009, a full-mouth periodontal examination was done as opposed to the partial-mouth examination that has previously been used. NHANES 2011–2012 found that 44.7% of adults aged 30 years and older had periodontitis, which is similar to the 47.2% found in the 2009–2010 survey [9]. The prevalence of periodontitis was higher among those living below the federal poverty line, those who had less than a high school education, and those who were current smokers [9]. Using disability adjusted life years (DALYs), it was found that oral health conditions contributed to 224 years of healthy living lost per 100,000 people [8]. 

## 3. Dentition and Food Choices

The association between periodontal disease and/or tooth loss and increased risk of chronic disease can, at least partially, be related to diet (Table 1). When dietary intakes were evaluated using 24-h recall questionnaires or food frequency questionnaires, it was found that individuals who are missing teeth tend to consume fewer servings of fruits and vegetables, less fiber, and more cholesterol [10,11,12]. This may be because missing teeth can limit an individual’s food choices. It can also impact the method they choose to cook their foods. Foods that have a more healthful nutrient profile are often more difficult to masticate, particularly for older adults. Examples include fruits, raw vegetables, and meats.

A study of Finnish adults aged 30 years and older included 7190 dental patients and assessed dietary intakes using a 24-h dietary intake recall questionnaire [13]. These patients were divided based on their dental status of dentate or edentulous. The dentate group was further divided into those who had removable prosthetics and those who did not. The edentulous group was divided into those who had both an upper and lower denture, which was considered adequate rehabilitation and those who had only one denture (either upper or lower) or no dentures, which was considered inadequate rehabilitation. The dentate population ate more fruits and vegetables, including root vegetables, than the edentulous population. Among the dentate group, having a higher number of natural teeth increased the probability of the participant eating fruits, vegetables (including root vegetables), and meat. Analysis of the edentulous group showed that having adequate rehabilitation was positively associated with eating more fruits, vegetables, and root vegetables. No difference was found between the adequacy of dental rehabilitation and the likelihood that they consumed an easy to chew food such as porridge within either the dentate or edentulous group [13]. It can therefore be inferred that individuals who lack adequate rehabilitation for their missing teeth avoid foods that are harder to chew such as fruits and raw vegetables. The impaired ability to eat fruits and raw vegetables could lead to an increased risk of chronic disease due to lower intakes of polyphenols that may act as antioxidants. Although it varies by vegetable and cooking method, the polyphenol content of some cooked vegetables (i.e., carrots, broccoli) is lower than the raw form [14]. Thus, the antioxidant capacity of vegetables can also be altered in the cooking process changing their ability to scavenge free radicals [14]. Some vegetables are best consumed raw to get the full antioxidant benefit, but consuming these foods can be difficult when the dentition is compromised. 

The relationship between dentition status and food choice was examined for adults aged 65 years and older who participated in the British National Diet and Nutrition Survey [15]. An oral examination was performed for each participant. The number of contact points between opposing teeth (top and bottom) were recorded for dentate individuals (anyone with some remaining natural teeth), as there is evidence to suggest this affects one’s ability to chew effectively [16]. All the edentulous participants had dentures. Data on food choice was gathered by giving participants a list of sixteen food items that ranged in masticatory difficulty. Examples include bread, carrots and steak. Participants were asked to indicate the level of difficulty they would experience when eating the specific food item. Specifically, they were asked whether they could eat it easily, with some difficulty, or not at all. Within the dentate group, 28% of participants reported they would have difficulty eating apples or would not be able to eat apples at all. That number increased to 50% within the edentulous group. The differences between the dentate compared to the edentulous groups’ anticipated ability to eat tomatoes, raw carrots, apples, and nuts were significant, while the anticipated ability to eat foods such as bread, toast, cheese, roasted potatoes, cooked greens, and chocolate did not differ significantly among groups. It was also found that number of natural teeth remaining affected the anticipated ability to eat certain foods within the dentate group. Of the participants with one to 10 teeth, 45% reported that they would have difficulty eating or would not be able to eat apples whereas of the participants with 21 or more teeth, only 12% reported anticipated difficulty or inability to eat apples. A total of 26% of participants with 11–20 teeth reported they would have difficulty or be unable to eat apples [15]. Apple consumption is associated with health benefits including decreased risk of obesity and cardiovascular disease [17,18]. The decreased risk of cardiovascular disease is often attributed to their high fiber and polyphenol content [18]. Ability to eat other fruits and vegetables of similar texture can be inferred by ability to eat apples. 

A longitudinal survey of 30,000 participants investigated dietary intake of those who lost five or more teeth compared to those who lost no teeth during a four-year follow up [10]. A diet questionnaire was used to gather intake data about 131 foods and supplements [10]. The questionnaire revealed that individuals who lost teeth significantly reduced their intake of apples and pears in the follow-up period. Similar to other studies, the intake of pears, apples, and carrots increased as the number of teeth increased [10]. Knowing that dental status affects an individual’s ability to eat certain nutritious foods such as raw vegetables or various fruits and even some meats, there is concern that people with inadequate dentition can be at risk for nutritional deficiencies. They can also be more likely to consume foods that are softer in texture, but often tend to be higher in saturated fat and cholesterol. For example, edentulous participants in the longitudinal study consumed more saturated fat, cholesterol, and had a higher caloric intake than participants with 25 or more teeth [10]. One study has shown that 86% of individuals with 20 or more teeth allowed them to consume apples, carrots and steaks (cooked as “well-done”) with ease [15]. It has also been suggested that a better way to assess chewing ability is the number of posterior occluding pairs (POPs) that an individual has. POPs are the number of molars or premolars where the opposing mandibular and maxillary teeth are present [19]. Individuals with five pairs of POPs, out of a possible eight, were able to consume a wider variety of nutritious foods, i.e., fruit and raw vegetables, which resulted in a higher quality diet based on the Healthy Eating Index (HEI) [11]. Individuals with fewer than five POPs were more likely to avoid certain foods and eat a less varied and nutritious diet. 

While it has been shown that missing natural teeth limits food choice because people avoid foods that they find difficult to consume, it has not been well established if these limitations lead to specific nutrient deficiencies. The evidence in support of this, however, is growing. In a study of male health professionals that studied food and nutrient intake via a questionnaire, edentulous participants consumed significantly fewer vegetables and dietary fiber than their counterparts with 25 or more teeth [10]. The edentulous participants’ intake of carotenes and crude fiber was also significantly lower, while total caloric intake, cholesterol, and saturated fat intake were significantly higher than those with 25 or more teeth. This highlights the fact that diet quality differs between these groups. The results of the questionnaire showed that there was no significant difference between groups for total fruit or vitamin C intake [10]. It is possible that because of their higher energy intake, edentulous individuals are getting most of the nutrients they need, but they are getting them from less healthful foods. 

To gain a more accurate sense of nutrients consumed, serum markers of nutrient status can be measured and generally provide more accurate information than a dietary record. A study of participants aged 65 and older included a four-day food diary in addition to blood and urine analyses as a surrogate measure of nutrient intake [20]. The sample consisted of 407 dentate individuals and 346 edentulous participants (endentulous participants all had dentures). Analysis of the food diaries showed that edentulous participants consumed less protein, intrinsic sugars, milk sugars, fiber, calcium, non-heme iron, riboflavin, thiamin, niacin, pantothenic acid, vitamin E, and vitamin C. Blood and urine analyses revealed that only plasma ascorbate and plasma retinol were statistically different between the dentate and edentulous participants. When the food diaries for the dentate individuals were analyzed based on number of teeth, those with more teeth reported higher intake of protein, fat, and carbohydrate as well as fiber, intrinsic sugar, milk sugar, calcium, non-heme iron, pantothenic acid, vitamin C, and vitamin E. Intake of these nutrients was significantly associated with number of POPs, as were vitamin A, thiamin, riboflavin, and niacin. Only plasma ascorbate was significantly associated with the number of teeth remaining; those with more teeth had higher plasma ascorbate levels. Plasma retinol was lower in the edentulous group than the dentate group, but was not associated with number of teeth remaining or POPs [20]. Individuals who are missing teeth might not be at as high of a risk for deficiencies of specific nutrients as previously thought based on biochemical measures, but some nutrients, such as beta-carotene, were not measured.

A similar study was conducted using data from the third National Health and Nutrition Examination Survey (NHANES III) in which dietary intake was collected using a 24-h recall method [11]. Serum levels of vitamin C, vitamin E, folate, and beta-carotene were selected as indicators of nutritional status. Carotenes, folacin, and ascorbic acid were significantly lower for people with one to four POPs than people with five to eight POPs. Vitamin A was significantly lower for edentulous participants and participants who wore full dentures, and dietary fiber was highest for those with five to eight POPs and lowest in the edentulous group. When serum levels of these nutrients were measured, it was found that only beta-carotene and vitamin C were significantly associated with number of POPs. There was a positive relationship; a higher number of POPs was associated with a higher serum level of vitamin C and beta-carotene. Serum folate was lower in denture wearers than it was in people with five to eight POPs [11]. In both aforementioned studies, vitamin C status was associated with number of teeth or POPs remaining [11,20]. One possible explanation for this is that many of the foods that are good sources of vitamin C—broccoli, brussels sprouts and carrots—must be cooked and softened to allow consumption by people with missing teeth. Depending on the method of cooking, this can decrease the amount of vitamin C available by up to 38% [21].

## 4. Pervasiveness of Fear and Anxiety toward Dental Treatment and Dental Avoidance Behaviours

Among Canadian adults, the prevalence of dental anxiety ranges from 4.4% to 16.4% [22]. The prevalence among Australian adults was similar with 16.1% of a random sample reporting high dental fear [23]. In both studies, females were more likely to report having high dental fear than males [22,23]. It is not well understood why females have higher dental fear. In a survey looking at attitudes toward dental fear, women were shown to fear pain more than men [24]. A study that aimed to understand gender differences in pain expectations before surgery and the memory of pain following surgery found that women had significantly greater anxiety than men before and after surgery. Interestingly, although women expressed greater anxiety, they expected less pain prior to the periodontal treatment than men [25]. The gender differences of reported anxiety or fear are often attributed to societal expectations for men to be tough, which could lead to them underreporting their true fear and anxiety toward dental treatment. One study sought to show that societal expectations played a role in the reporting of dental fear between men and women by changing the wording “fear” with “dread” [26]. They hypothesized that men would admit feeling dread toward various dental treatments because dread is more socially acceptable than fear. This was partially supported in that they did find men were more likely to express dread than fear, but it was not isolated to men as women were also more likely to express dread than fear [26]. In the Canadian sample, there were no age differences found, but in the Australian sample, those aged 40 to 64 years were more likely to report high fear of dental treatment [22,23]. The two studies grouped patients by age using different cut points; this could account for the different findings. For instance, the Canadian sample was divided into six age groups (18 to 24 years, 25 to 34 years, 35 to 44 years, 45 to 54 years, 55 to 64 years, 65 years and older) while the Australian sample was divided into seven age groups that included a broader range of ages (under 13 years, 13 to 17 years, 18 to 24 years, 25 to 39 years, 40 to 64 years, 65 to 79 years, 80 years and older). 

High dental fear is a major reason why a patient may choose to miss and cancel appointments or to avoid dental appointments. When asked about why they had avoided the dentist in the past year, one study found that fear of dental treatment was the reason given by 7.8% of participants [22]. When patients were categorized as having a high level of dental fear versus those with low or no dental fear, those in the high dental fear group reported missing, cancelling, or avoiding the dentist significantly more than those in the low or no fear group [22]. Although fear is a major contributor to the reasons people avoid dental treatment it should be noted that it is not the only reason cited for not seeking dental treatment. Other common reasons people give for not seeking dental treatment include a lack of time, the cost of treatment, the feeling that treatment was not required, the concern about the pain associated with treatment, and a dislike of dentists [27,28]. 

Several studies have reported main factors that lead to avoiding or delaying dental treatment. In one study, reasons for avoiding the dentist in the past year included teeth had not been bothering them (40.7%), cost (30.7%), lack of time (14.9%), fear of dental treatment (7.8%), and other reasons (5.9%) [22]. When young adults (mean age 23.2 ± 4.0 years) were surveyed regarding their dental care practices, 36% of respondents reported “lack of time” as a reason for irregular visits [28]. A total of 34.1% reported that they did not have regular attendance because they felt that treatment was not needed, while 16.6% reported that cost of treatment limited their attendance, and 13.1% reported that fear kept them from the dentist [28]. Among the participants, those who had regular dental attendance reported lower dental anxiety than those who had irregular attendance (20.9% versus 79.1%) [28]. A different study divided patients into high and low dental anxiety groups based on responses to the Index of Dental Anxiety and Fear (IDAF-4C) [27]. All of the patients were asked if they were currently avoiding the dentist and the reason behind their avoidance. Patients in both the high and low dental anxiety groups reported cost as the most common reason for avoidance (72.5% versus 70.0%, respectively). In the high dental anxiety group, the next most common reasons for avoidance included fear or anxiety (55.6%), dislike of the dentist (41.2%), and concern about the pain or having an unpleasant experience (41.1%) [27]. In the low dental anxiety group, the second most common reason for avoidance following cost was lack of time (38.8%) [27]. There are a number of factors that contribute to a patient’s anxiety leading up to a dental visit, with fear of the pain associated with treatment as a primary cause, which can ultimately lead to avoidance of dental treatment. 

Dental fear can lead to long-term avoidance of visiting a dentist. More patients who had not been to the dentist in the previous 10 years had more dental fear than those who had been to the dentist within the previous 12 months [23]. Avoiding treatment can reinforce the fear of dental treatment because it often results in patients seeking dental treatment only when the oral health condition has deteriorated to such a degree that intensive treatment is required. In a study where patients reported avoiding the dentist for an average of 7.5 years (ranged from 0 to 40 years), it was found that the number of years an individual had avoided the dentist was correlated with poorer oral health [29]. Fear of dental treatment can also be reinforced when patients with high dental anxiety only seek treatment on a problem-oriented basis because a delay in seeking treatment can lead to more complicated clinical situations that might have been prevented with earlier intervention [27]. 

## 5. Impact of Fear and Anxiety on Periodontal Treatment 

In addition to general oral health and visits to the general dentist, some studies have investigated the impact of fear and anxiety on preventing patients from seeking periodontal treatment. Desire for anesthesia can serve as an indicator of dental fear or anxiety. Using this premise, it is possible that individuals who desire sedation or general anesthesia experience the greatest negative perception toward periodontal surgery. In the surveyed population, preference for sedation was highest for periodontal surgery (68.2%) compared to endodontic procedures (54.7%) or extraction (46.5%) [22]. 

Patients who have greater anxiety regarding a periodontal procedure ultimately experience a greater amount of pain compared to patients with low levels of anxiety [30]. With respect to dental implant surgery, anxiety was the strongest predictor of the amount of pain a patient would experience following surgery [30]. Similarly, another study found that patients with high anxiety prior to periodontal surgery experienced significantly more pain than those with low anxiety prior to periodontal surgery [31]. 

## 6. Strategies for Reducing Dental Fear and Anxiety

It is critical to remove as many of the barriers to dental treatment as possible so patients will seek required dental treatment. Receiving the appropriate dental treatment in a timely manner should help individuals to maintain their natural teeth or to receive dental implants so that they are able to eat a healthy and varied diet. As shown in Table 2, there are a variety of potential strategies that have been used to manage anxiety in the dental setting.

Patient concerns regarding dental visits differ greatly based on how they perceive the dentist and dental treatment. Some psychological aspects of dental treatment are the cause of a patient’s nervousness. Previous reports have shown that patients report more anxiety toward dental treatment when they have previously had a painful dental experience [27,32]. For example, it was found that the severity of a previous negative experience influenced an individual with high dental anxiety to avoid dental treatment [27]. Negative experiences that were explored included pain, discomfort, gagging, fainting or feeling light-headed, embarrassment, and having a personal problem with the dentist. Among patients with high dental anxiety, those who had previously fainted or felt embarrassment because of dental treatment had a significantly greater chance of dental avoidance. For those with low dental anxiety, there was a significant relationship between a previous experience involving pain or fainting that they considered to be strongly negative and current dental avoidance [27]. It has also been reported that some patients feel anxious when there is a perceived loss of control in their treatment [32,33]. It is important in these situations that the dental professional is able to identify nervous patients and effectively communicate with them. This helps build trust between the patient and the dental professional. In turn, this can reduce anxiety and help the patient to feel more in control of their treatment [32]. Effective communication between the dental staff and the patient is essential for building trust in the relationship and putting the patient at ease. According to a study of a group of patients who had high dental anxiety but who managed to overcome their anxiety to seek regular dental care, a foundation of trust was key to making this possible. Communication was identified as a pillar in building the trust in this relationship [34].

Some aspects of dental treatment that trigger anxiety are physically uncomfortable experiences such as bad tastes, receiving the local anesthetic or having too much fluid in the mouth [31]. A study of negative experiences at consecutive surgeries showed there was a poor correlation between the specific experiences that were causing discomfort. It was hypothesized that this was because if a patient had an uncomfortable experience during the first surgery, then the patient was able to communicate this to the surgeon to avoid the negative experience in the following visit [31]. This is an encouraging finding, but again it emphasizes the need for effective communication between the patient and the clinician. 

Creating an environment where a patient feels at ease can also help to reduce fear and anxiety. The reception area of dental offices is an opportunity to do this. To determine if aromatherapy could reduce anxiety in the reception area, patients were divided into four groups: a control with no intervention, a group who listened to music, a group exposed to the scent of orange, and a group exposed to the scent of lavender. Significant differences were found between the control and the orange scent group and between the control and the lavender scent group, but not between the control and the music group. Those in the aromatherapy groups reported less anxiety and a greater feeling of calmness [35]. While there was no benefit with the music intervention [35], there is some evidence to show that music therapy can help reduce anxiety, albeit not as effectively as other methods [36]. A brief relaxation technique, music therapy, and a control were compared on their efficacy for decreasing dental anxiety. Anxiety decreased in both the relaxation and music groups compared to the control, but anxiety in the relaxation group was also significantly lower than the music group. The reduction in anxiety in the relaxation group was the only one deemed clinically relevant [36]. 

Functional relaxation is an example of a behavioural therapy that has been explored to reduce anxiety; other examples include distraction using virtual reality and hypnosis. The virtual reality intervention included various relaxing natural environments such as a beach, a forest, or mountains that the patient could navigate. Patients experienced 5 min of treatment without the distraction then 5 min of treatment with the distraction. Self-evaluation and physiological measures supported the notion than distraction helps reduce anxiety in a dental setting [37]. To look at the effects of hypnosis, patients were assigned to either the treatment only group or treatment with hypnosis. Hypnosis was achieved by having patients listen to a recording. After treatment, patients from both groups were asked to rate their anxiety during treatment and after treatment. Those in the hypnosis group reported having lower levels of anxiety during treatment [38].

If a patient experiences greater levels of fear or anxiety, to the extent where communication and giving the patient a sense of control in the treatment is not sufficient to allow them to feel at ease, sedation or general anesthesia is a viable alternative [22]. A total of 31.1% of patients with high dental fear were ‘definitely interested’ in sedation, another 54.1% of those patients indicated they ‘might be interested’ depending on cost [22]. Interestingly, even some patients who reported low levels of dental fear were interested in sedation [22]. Another study examined the differences in dental anxiety between a group who had local anesthetic alone and a group who had local anesthetic combined with conscious sedation. Patients who had sedation plus local anesthetic reported less pain when they arrived before the surgery and recalled (1-month post-surgery) less pain and a lower level of predicted and recalled anxiety. It was suggested that if individuals recall less pain and anxiety following treatment, they are more likely to seek treatment in the future [39].

It is important for a clinician to identify patients who are anxious about treatment so the clinician is able to make them feel comfortable about the process. Encouraging dentally anxious patients to ask questions regarding their procedure might make them feel at ease, and thus more likely to seek the treatment they need to restore oral health and allow them to live a more healthful lifestyle overall. Clearly, finding effective strategies to eliminate or manage anxiety in a dental setting is a key to breaking the cycle shown in Figure 1. Further study is needed to develop such strategies.

## 7. Conclusions 

The literature demonstrates that oral health status affects food and nutrient intake. It can influence the perceived ease with which individuals eat different foods and there is some evidence that individuals with fewer teeth avoid foods that can be considered difficult to chew [10,15]. Individuals with fewer teeth have been found to have lower intake or status of some nutrients, such as vitamin C, compared to individuals with more teeth [11]. It can be concluded that fear is a reason patients avoid dental treatment and some evidence suggests that periodontal surgery might cause patients the greatest anxiety compared to other types of dental surgery [22,28]. This dental fear and anxiety can also lead to avoiding periodontal surgery. Current knowledge gaps include identifying how the nutritional status of patients with dental anxiety compares to patients who do not suffer from dental anxiety and whether restoring the dentition leads to more variety in food choice. It is reasonable that if the cycle can be interrupted through further understanding of this complex relationship, patients are likely to live more healthfully and the patient can be made less apprehensive about the dental experience. 

## Figures and Tables

**Table 1:** Summary of findings regarding dentition and food choices.

Dentition Status	Dietary Assessment	Findings	Reference
Number of teeth at the beginning and end of the study were quantified. Some analyses compared those who lost no teeth and five or more teeth during the 4-year follow-up. Other analyses used total number of teeth.	A food frequency questionnaire that included 131 foods and supplements was sent to all participants.	Comparison between edentulous group and those with 25 or more teeth revealed that the intake of vegetables, fiber, and carotene was lower in the edentulous group, while caloric intake, saturated fat, and cholesterol were higher. Participants who lost five or more teeth tended to consume less fruits and vegetables; specifically apples and pears, than those who had no tooth loss.	[10]
Number of posterior occluding pairs (POPs), number of posterior teeth, and total number of teeth was recorded	A 24-h dietary recall questionnaire used for dietary intake; serum, blood, and urine was used to measure nutrient levels.	Participants with fewer than five POPs and those who wore dentures had lower Healthy Eating Index (HEI) scores. Serum vitamin C was positively related to number of POPs and number of teeth when it was dichotomized to fewer than 18 teeth and more than 18 teeth.	[11]
Dentate individuals without dentures	Food frequency questionnaire and 24-h dietary recall.	Intake of carrots and tossed salads consumed per month were lower in all groups missing teeth (less than 28 teeth). Serum beta-carotene was lower in participants with fewer than 21 teeth compared to those with full dentition. Serum vitamin C levels decreased as number of teeth decreased.	[12]
Dentate (subdivided into those with removable dentures and those without). Edentulous (subdivided into those with adequate rehabilitation- upper and lower dentures and those with inadequate rehabilitation- one or no dentures).	A 24-h self-administered diet questionnaire. Intake was measured on an all-or-none basis regarding whether they had consumed a specific food in the previous day or not.	The dentate group consumed more fruits and vegetables, including root vegetables, than edentulous group. Among the dentate group, presence of removable dentures and a higher number of teeth resulted in higher consumption of fruits and vegetables, including root vegetables, and meat. Among the edentulous group, adequate rehabilitation resulted in higher consumption of fruits and vegetables, including root vegetables, and meat.	[13]
Dentate (number of POPs was recorded). Edentulous (all had dentures).	Participants responded with the amount of difficulty they would experience if they attempted to eat 16 listed food items.	A total of 28% of the dentate group indicated they would have difficulty eating apples. They would also have difficulty eating nuts, steaks, raw carrots. Higher number of POPs resulted in lower anticipated difficulty eating certain foods such as apples. A total of 50% of the edentulous group would have difficulty eating apples. They would also have more difficulty eating tomatoes, carrots, apples, and nuts than dentate individuals.	[15]
Number of natural teeth, POPs, and if they wear partial dentures was recorded for dentate participants	A 4-day weighted diary of foods and drinks consumed.	Dentate participants had higher intakes of protein, fiber, intrinsic and milk sugars, calcium, non-heme iron, thiamin, riboflavin, niacin, pantothenic acid, vitamin C, and vitamin E than edentulous participants. Among the dentate participants, those with a greater number of teeth had higher intakes of energy, protein, fat, carbohydrates, intrinsic and milk sugars, non-heme iron, calcium, pantothenic acid, vitamin C, and vitamin E.	[20]

**Table 2:** Strategies to manage anxiety in a dental setting.

Strategy	Evidence of Efficacy
Communication	Effective communication between the dental staff and the patient is essential for building trust in the relationship and putting the patient at ease [34].
Sedation or General Anesthesia	Sedation can lower a patient’s current state of pain and recalled pain. It can also lower predicted and recalled anxiety [39].
Relaxation	When patients were given instructions on functional relaxation and performed it during their treatment, it significantly lowered their anxiety [36].
Distraction	Virtual reality was tested to determine if distraction from the dental treatment reduced anxiety. Patients’ self-evaluation and physiological measures showed that distraction helps reduce anxiety in a dental setting [37].
Hypnosis	Patients who were hypnotized reported having lower levels of anxiety during treatment than those who were not [38].
Aromatherapy	Patients in the aromatherapy groups reported less anxiety and a greater feeling of calmness [35].

## Abbreviations

The following abbreviations are used in this manuscript:

CVD	Cardiovascular disease
DALY	Disability adjusted life year
HEI	Healthy Eating Index
IDAF-4C	Index of Dental Anxiety and Fear-4C
NHANES	National Health and Nutrition Examination Survey
POPs	Posterior occluding pairs
